# Identification of Various Recombinants in a Patient Coinfected With the Different SARS‐CoV‐2 Variants

**DOI:** 10.1111/irv.13340

**Published:** 2024-06-18

**Authors:** Yusuke Sayama, Akie Sakagami, Michiko Okamoto, Masahiro Sakamoto, Hikari Koizumi, Yoko Kimura, Clyde Dapat, Mayuko Saito, Yuko Suzuki, Mie Sasaki, Naoko Sugawara, Hitoshi Oshitani

**Affiliations:** ^1^ Department of Virology Tohoku University of Graduate School of Medicine Sendai Miyagi Japan; ^2^ Department of Microbiology Miyagi Prefectural Institute of Public Health and Environment Sendai Miyagi Japan

**Keywords:** coinfection, recombination, SARS‐CoV‐2, spike gene, viral quasispecies

## Abstract

**Background:**

Viral recombination that occurs by exchanging genetic materials between two viral genomes coinfecting the same host cells is associated with the emergence of new viruses with different virulence. Herein, we detected a patient coinfected with severe acute respiratory syndrome coronavirus 2 (SARS‐CoV‐2) Delta and Omicron variants and identified various recombinants in the SARS‐CoV‐2 full‐length spike gene using long‐read and Sanger sequencing.

**Methods:**

Samples from five patients in Japan with household transmission of coronavirus disease 2019 (COVID‐19) were analyzed using molecular assays for detection and identification of SARS‐CoV‐2. Whole‐genome sequencing was conducted using multiplex PCR with short‐read sequencing.

**Results:**

Among the five SARS‐CoV‐2‐positive patients, the mutation‐specific assay identified the Delta variant in three, the Omicron variant in one, and an undetermined in one. The undermined patient was identified as Delta using whole‐genome sequencing, but samples showed a mixed population of Delta and Omicron variants. This patient was analyzed for viral quasispecies by long‐read and Sanger sequencing using a full‐length spike gene amplicon. In addition to the Delta and Omicron sequences, the viral quasispecies analysis identified nine different genetic recombinant sequences with various breakpoints between Delta and Omicron sequences. The nine detected recombinant sequences in the spike gene showed over 99% identity with viruses that were detected during the Delta and Omicron cocirculation period from the United States and Europe.

**Conclusions:**

This study demonstrates that patients coinfected with different SARS‐CoV‐2 variants can generate various viral recombinants and that various recombinant viruses may be produced during the cocirculation of different variants.

**Abbreviations**
CCS: circular‐consensus sequencing
COVID‐19: coronavirus disease 2019
SARS‐CoV‐2: severe acute respiratory syndrome coronavirus 2
SNP: single‐nucleotide polymorphism
VOC: variant of concern
WGS: whole‐genome sequencing

## 1 Introduction

Viruses undergo genetic changes through several mechanisms, such as point mutation and recombination. Viral recombination, which increases genetic diversity and accelerates adaptation in viral populations, is a commonly observed evolutionary feature among both DNA and RNA viruses [1, 2]. Recombination occurs when at least two viral genomes coinfect the same host cell and exchange their genetic fragments. Viral recombination leads to the expansion of viral host ranges, emergence of new viral strains, alteration of transmission vector specificities, increases in virulence and pathogenesis, modification of tissue tropisms, evasion of host immunity, and evolution of resistance to antivirals [1, 3, 4]. Viral recombination has been observed in several viruses, including members of the *Coronaviridae* family, to which the human coronaviruses belong [5].

Coronavirus disease 2019 (COVID‐19), caused by severe acute respiratory syndrome coronavirus 2 (SARS‐CoV‐2), was first detected in Wuhan, China, in December 2019 [6]. Since then, SARS‐CoV‐2 has evolved into phylogenetically distinct lineages, some of which have been designated as variants of concern (VOC) [7, 8]. These variants differ in terms of transmissibility, virulence, and immune escape capability against humoral immunity [9, 10]. Particularly, the SARS‐CoV‐2 spike gene is crucial for vaccine development and treatment using monoclonal antibodies [11].

SARS‐CoV‐2 recombination has been identified in some strains through whole‐genome sequencing (WGS) [12, 13]. In addition, the number of infections with SARS‐CoV‐2 recombinant viruses, including XBB.1.5, XBB.1.16, and EG.5.1, has increased worldwide from late 2022 (GISAID, https://gisaid.org/). However, only a few studies have analyzed SARS‐CoV‐2 recombination in patients coinfected with different variants. Particularly, the identification of viral quasispecies using long‐read sequencing has been poorly documented [14, 15]. Recombinant viruses play a significance role in the evolution of the novel viruses, contributing to the generation of genetic diversity and the emergence of novel viral strains. Therefore, it is important to analyze recombinants in patients coinfected with different SARS‐CoV‐2 variants. Thus, this study aimed to identify recombination sequences by long‐read and Sanger sequencing using a full‐length spike gene amplicon from a patient coinfected with the Delta and Omicron variants. Our findings provide novel insights into recombination events in SARS‐CoV‐2.

## 2 Material and Methods

### 2.1 Sample Collection, Screening Tests, and WGS

In February 2022, nasopharyngeal swabs were collected from 12 patients in Miyagi Prefecture, Japan, with COVID‐19 in the household. Laboratory molecular diagnosis confirmed that 11 of the 12 patients were positive for SARS‐CoV‐2. We conducted further studies using samples from 5 of the 11 patients with SARS‐CoV‐2 infection. Samples were not available from the other six patients. RNA was extracted using RNA extraction kits (Promega Maxwell RSC Viral Total Nucleic Acid Purification kit, Promega, Madison, WI, USA; QIAamp Viral RNA Mini Kit, Qiagen, Hilden, Germany). The extracted RNA was screened for SARS‐CoV‐2 detection using a reverse‐transcription real‐time PCR assay targeting the SARS‐CoV‐2 nucleocapsid (N) gene [16]. Positive samples were further tested for VOC‐specific amino‐acid substitution by targeting the single nucleotide polymorphism (SNP) spike L452R [17]. Amplification and genotyping results were analyzed using QuantStudio 5 (ThermoFisher, Waltham, MA, USA). Complementary (c) DNA was synthesized using a LunaScript RT Super Mix kit (New England Biolabs, Ipswich, MA, USA), and WGS of SARS‐CoV‐2 was performed by multiplexed PCR amplification using ItokawaK primer set ver_N4 [18]. PCR products were purified and subjected to Illumina library construction using Illumina DNA Prep Tagmentation and Nextera DNA CD indices (Illumina, San Diego, CA, USA). The MiniSeq platform (Illumina) was used to sequence the indexed libraries using the MiniSeq High Output Reagent Kit (300 cycles). Data analyses were processed using the Dragen Lineage Pipeline 3.5.3 (Illumina), with each parameter set to default. This pipeline provided the percentage of non‐N bases (coverage ≥ 10), median coverage, Pango lineage, Clade, and FASTA consensus sequences.

### 2.2 Deep Sequencing Using Long‐Read and Sanger Sequencing

cDNA was amplified using nested PCR targeting of the SARS‐CoV‐2 full‐length spike gene with PrimeSTAR MAX (TaKaRa Bio, Shiga, Japan). The primer set used for the first PCR was CoV‐2_S_Full_Fw1: 5′‐AAGAAGGTCAAATCAATGATATGA‐3′ and CoV‐2_S_Full_Rv1: 5′ ‐GCGCGAACAAAATCTGAAGGAG‐3′. The primer set used for the nested PCR was CoV‐2_S_Full_Fw2: 5′‐ACAACAGAGTTGTTATTTCTAGTGATG‐3′ and CoV‐2_S_Full_Rv2: 5′‐CAGTTCCAATTGTGAAGATTCTC‐3′. The amplified PCR products were observed by gel electrophoresis and purified using a PCR purification kit (Qiagen). Amplicons were tagged with each PCR primer using KAPA HiFi HotStart Ready Mix (Nippon Genetics, Tokyo, Japan), adjusted for libraries using PacBio Barcoded Universal Primers for Multiplexing Amplicons (PacBio, CA, USA), and sequenced using Binding Kit 2.2 (PacBio) by Sequel IIe (PacBio). Long‐read sequencing data were generated using circular‐consensus sequencing (CCS). Reads of less than 20 CCS as the average quality value per one read were removed using SMRT Link (PacBio, v.10.1.0.119528). Chimera and noise fragments were removed from the data using the Qiime 2 pipeline (https://qiime2.org/, ver. 2020.11.1 and ver. 2022.2) with Divisive Amplicon Denoising Algorithm 2 (DADA2), and each fragment was generated. Moreover, the PCR product of the SARS‐CoV‐2 full‐length spike gene was cloned using Mighty TA‐cloning Reagent Set for PrimeSTAR (TaKaRa). Clones were sequenced using BigDye chemistry via genetic analyzers (ABI 3730xl and 3500xL, ThermoFisher Scientific). Putative recombinant analysis was examined by comparison of genetic similarity using the SimPlot program [19].

## 3 Results

### 3.1 Clinical Information of Patients

The five patients included in this study were 26–44 years old, immunocompetent, and unvaccinated for COVID‐19 (Table 1). They developed COVID‐19 symptoms between January 31 and February 2, 2022. Three (H540, H543, and H544) of the five patients required hospitalization.

**TABLE 1:** Demographic, clinical, and virological characteristics of the five patients in this study.

	Sample (ID)				
	1 (H540)	2 (H541)	3 (H542)	4 (H543)	5 (H544)
Age	34	27	26	44	34
Sex	Female	Female	Female	Male	Male
COVID‐19 vaccination	No	No	No	No	No
Date of onset	Feb 1, 2022	Feb 2, 2022	Feb 2, 2022	Jan 31, 2022	Feb 2, 2022
Clinical information					
Fever (> 37.5°C)	+	+	+	+	+
Cough	+	+	+	+	+
Difficulty breathing	−	+	−	+	−
Sore throat	+	−	−	+	+
Headache	+	+	+	+	+
Nasal discharge	−	+	−	−	−
Joint muscle ache	+	+	+	+	+
Diarrhea	−	−	−	+	+
Chill	+	−	−	+	+
General malaise	+	+	−	−	+
Taste disturbance, dysosmia	−	−	+	+	−
Hospitalization	+	−	−	+	+
Medical history	No	No	Buckwheat allergy	Hepatitis C 21 years previously	No
Smoker	Yes (20/day)	No	Yes (30/day)	Yes (20/day)	Yes (20/day)
Real‐time PCR: N gene (Ct)	21.9	22.1	21.5	25.3	27.3
SNP screening L452R	L (Omicron)	R (Delta)	Not determined	R (Delta)	R (Delta)
WGS					
WGS, % of non‐N bases (coverage ≥ 10×)	99.73%	99.72%	99.73%	99.73%	99.73%
Median coverage	1586	1279	1444	1104	1557
Lineage	BA.1.1.2	AY.29.2	AY29.2	AY.29.2	AY29.2
Clade (Nextclade)	21K	21J	21J	21J	21J
Conclusion	Omicron	Delta	Delta, Omicron, and Delta/Omicron recombinant	Delta	Delta
Accession numbers	DRR438334	DRR438335	DRR438336 and DRR438339	DRR438337	DRR438338

Abbreviations: SNP, single‐nucleotide polymorphism; WGS, whole‐genome sequencing.

### 3.2 Molecular Test and WGS Results

**FIGURE 1:**
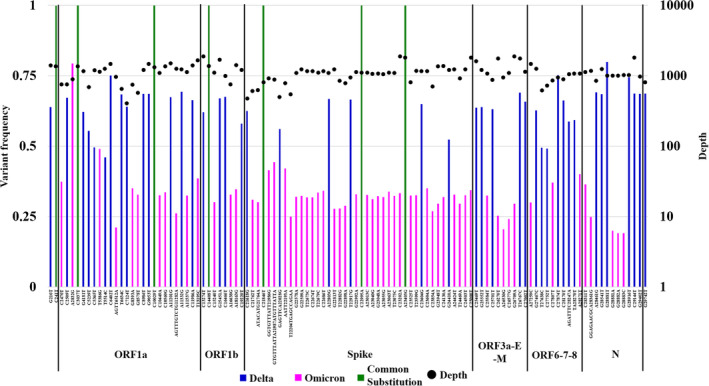
Representation of variant frequency of clade‐defining mutations of SARS‐CoV‐2, including Delta and Omicron variants, using whole‐genome sequencing via short‐read sequencing. Each clade‐defining mutation based on WT (Accession no. NC_045512) along the genome of the coinfected patient is shown. The variant allele frequency shows mutations on the left Y‐axis, and the depth of each genomic position is shown on the right Y‐axis. ORF, open reading frame.

We detected samples from the five patients (H540–H544) using PCR targeting of the N gene (Table 1). Using an SNP spike L452R assay, the Omicron variant was identified in one patient (H540), and the Delta variant was identified in three patients (H541, H543, and H544). However, the variant in the sample from the remaining patient (H542) could not be determined using the SNP PCR assay. SARS‐CoV‐2 whole‐genome analysis using short‐read sequencing yielded similar results in patient H540 (infected with Omicron variant BA.1.1.2, 21K) and patients H541, H543, and H544 (all infected with Delta variant AY29.2, 21J). Patient H542, in whom the variant was undetermined by the SNP PCR assay, was found to be detected with the Delta variant (AY29.2, 21J) using WGS. However, clade‐defining mutation analysis showed the presence of both Delta and Omicron variants in this patient (H542; Figure 1), suggesting coinfection with both variants. The Delta variant exhibited a higher population than the Omicron variant in the patient with coinfection.

### 3.3 Viral Quasispecies Analysis of the Full‐Length Spike Gene Using Long‐Read and Sanger Sequencing

**FIGURE 2:**
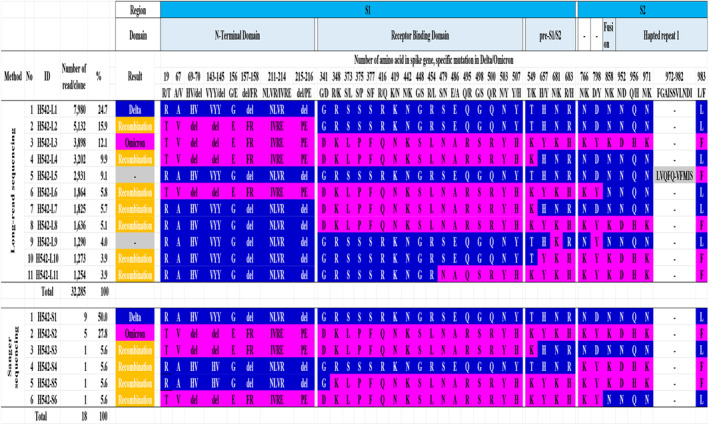
Description of 13 viral quasispecies amino acids identified via long‐read and Sanger sequencing using a full‐length spike gene amplicon. Each color is specific for a variant and insertion/deletion: blue (Delta), magenta (Omicron), and gray (insertion and deletion). H542‐L1 to L11 obtained based on long‐read sequencing. H542‐S1 to S6 obtained based on Sanger sequencing. The amino acids of H542‐L5 were located at 970–980 because of a deletion at 2904 and an insertion at 2939 of thymidine in the thymidine‐repeating region in the nucleotide.

**FIGURE 3:**
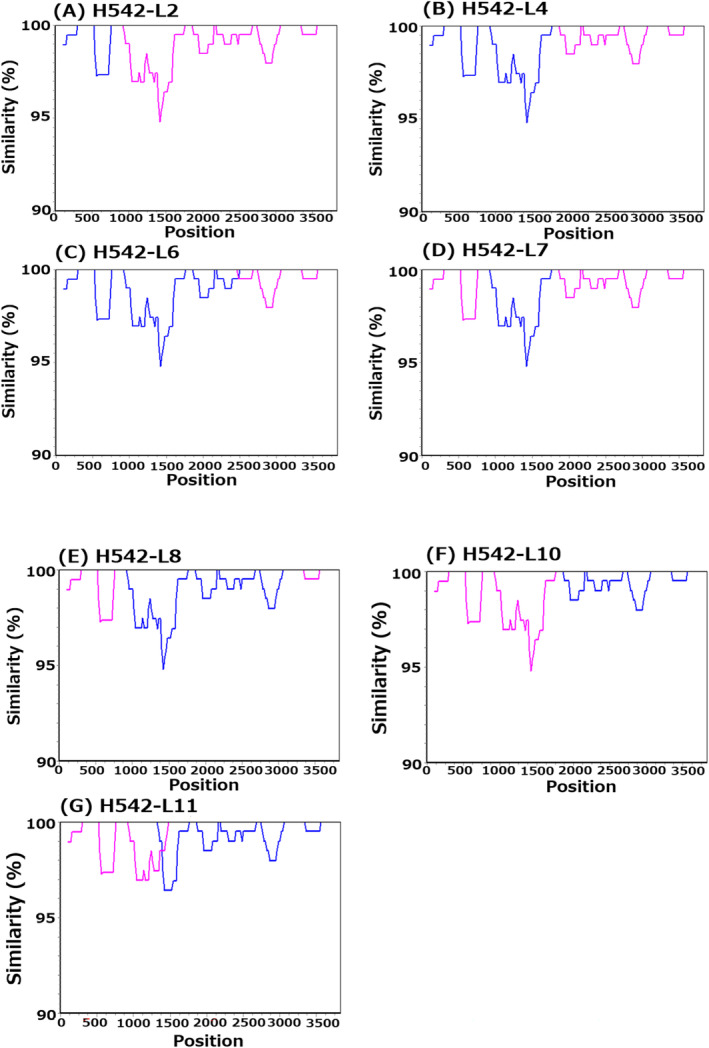
SimPlot analysis for putative SARS‐CoV‐2 recombinants. Comparisons of genetic similarity between recombinant and Delta (H542‐L1/blue) and Omicron (H542‐L3/magenta) sequences were made using the SimPlot software. The results are shown for the viral quasispecies sequences H542‐L2 (A), H542‐L4 (B), H542‐L6 (C), H542‐L7 (D), H542‐L8 (E), H542‐L10 (F), and H542‐L11 (G). The vertical axis represents the percent nucleotide sequence similarity between the putative recombinant and each strain used for comparison, and the horizontal axis shows the relative nucleotide position along the Spike gene. In each analysis, a window size of 200 nucleotides and the Kimura distance model (2‐parameter) were used.

Long‐read sequencing using a full‐length spike gene amplicon detected 11 viral quasispecies (H542‐L1–L11) from 32,285 reads in sample from the patient with Delta and Omicron coinfection (Figure 2). Sanger sequencing using the same amplicon also obtained a total number of 18 clones. The nucleotides and amino acids of viral quasispecies are shown in Figures S1 and S2. In long‐read sequencing, H542‐L1 (24.7%) and H542‐L3 (12.1%) were identified as Delta and Omicron sequences, respectively. Additionally, seven viral quasispecies were detected as recombinations with distinct recombination breakpoints and different populations using SimPlot analysis (Figure 3) and amino acid analyses (3.9%–15.9%). Among them, H542‐L8 (5.1%), H542‐L10 (3.9%), and H542‐L11 (3.9%) showed Delta–Omicron recombinant sequences, whereas H542‐L2 (15.9%), H542‐L4 (9.9%), and H542‐L6 (5.8%) showed Omicron–Delta sequences. H542‐L7 (5.7%) showed Delta–Omicron–Delta sequences in the full‐length spike gene. H542‐L5 (9.1%) showed different amino acids at 970–980 because of a deletion and insertion of thymine at positions 2904 and 2939 of the thymine‐repeating region in the nucleotide. H542‐L9 (4%) showed two Omicron‐specific point mutations in the Delta‐specific variant. Most recombinant sequence breakpoints were observed in the S1 region; however, H542‐L6 was the only viral quasispecies with a breakpoint in the S2 region. In Sanger sequencing, nine (50%) and five (27.8%) of the 18 clones were identified as Delta and Omicron sequences, respectively (Figures 2 and S3). Four clones (H542‐S3 to S6, 22.4%) were identified as recombinants. Two clones had the same fragments in amino acids as those obtained based on long‐read sequencing (H542‐L4 and H542‐S3; H542‐L6 and H542‐S6). In the remaining two (H542‐S4 and H542‐S5) clones, we detected different recombinant sequences that show Delta‐Omicron sequences in the full‐length spike gene. Finally, we conducted a Basic Local Alignment Search Tool (BLAST, https://blast.ncbi.nlm.nih.gov/Blast.cgi) analysis using each recombinant sequence obtained in this study. The detected recombinants showed a high level of identity (99.74%–99.99%) with virus strains that were detected during the Delta and Omicron cocirculation period from the United States and Germany (Table S1). These virus strains were detected as putative recombinants using SimPlot analysis (Figure S4).

## 4 Discussion

In this study, we identified Delta and Omicron sequences and at least nine distinct recombinant sequences in a patient coinfected with the SARS‐CoV‐2 Delta and Omicron variants. This coinfected patient was identified to be infected with the Delta variant by whole‐genome analysis using short‐read sequencing; however, the analysis also showed a mixed population of Delta and Omicron variants. Previous studies have reported 0.13%–0.25% detection rates of SARS‐CoV‐2 coinfection during the period of Delta and Omicron cocirculation [14, 15, 20]. The detection rates of recombinants among samples from patients with coinfection in these studies were 1/7 (14%) and 2/18 (11%) [14, 15], suggesting that the detection rate of patients with coinfection and recombinants is very low. In this study, among the 11 patients positive for SARS‐CoV‐2 infection, we could not analyze SARS‐CoV‐2 variants in six patients owing to the lack of available samples. One of the five patients in whom we conducted variant analysis was identified as having Delta and Omicron coinfection, which is a higher recombinant detection rate than that reported in previous studies. Further studies with larger sample sizes are required to analyze the frequency of coinfection in patients during periods of cocirculation of different SARS‐CoV‐2 variants and the recombinants generated in patients with coinfection.

In the present study, we identified one patient (H542) who was infected with the Delta and Omicron variants and various recombinant viruses. This patient, aged 26 and nonvaccinated for COVID‐19, showed fever (>37.5°C), cough, headache, joint muscle ache, and taste disturbance dysosmia. However, the patient was not hospitalized because, in Japan, samples were not used to determine the requirement for hospitalization according to the severity of illness at that time. Consequently, we could not analyze the severity of infection in the patients enrolled in this study. It was reported that patients infected with SARS‐CoV‐2 recombinant viruses showed low clinical severity until infection with the XD strain, a recombinant of the Delta and Omicron variants [21]. Thus, recombinant viruses originating from the Delta and Omicron variants may cause clinical symptoms with low severity.

This study identified two variants (Delta and Omicron) of SARS‐CoV‐2 and nine recombinants with distinct breakpoints using full‐length spike gene amplicons by long‐read and Sanger sequencing. Deep sequencing investigation of the viral population revealed that the Delta variant (in H542‐L1 and H542‐S1) was predominant. This result is consistent with the WGS results, which showed that the viral population of the patient with coinfection comprised a higher number of Delta than Omicron sequences. Other studies of patients with coinfection with different SARS‐CoV‐2 variants have also found that the size of the viral population of each variant differed [22]. The virus population in patients with coinfection might be determined by several factors, such as host immunocompetence, vaccination history, and virus phenotype. This study identified nine recombinants in the patient with coinfection. A previous study reported two types of recombination sequences in a patient with Delta–Omicron coinfection using long‐read sequencing of the partial spike gene [14]. Our results confirmed that patients with coinfection can generate various types of recombination sequences. The patient with coinfection in this study had not been vaccinated against SARS‐CoV‐2. In unvaccinated patients, SARS‐CoV‐2 shows high within‐host diversity [23]. Therefore, various recombinants might have been generated in the patient with coinfection in this study. We further detected recombinants not detected by long‐read sequencing by Sanger sequencing. Sanger sequencing is only able to detect minority variants at frequencies between 10% and 40%, has limited power to sequence genomes, and involves high costs compared with next generation sequencing, including long‐read sequencing [24]. In contrast, for long‐read sequencing, many cycles of amplification are needed to add tagged PCR amplicons using PCR. Analysis of recombinant by both methods using samples from coinfected individuals is not well understood. Thus, future analyses using samples from coinfected patients should be performed to examine and ascertain the efficacy of these methods. This study identified that the nine recombinants with various breakpoints (at 216–348, 454–479, 549–657, 683–766, and 798–858) in the spike gene amino acids. However, the breakpoints detected in this study differed from those in XD. SARS‐CoV‐2 recombination breakpoints have been reported to occur disproportionately in the 3′ region of the genome containing the spike gene [25]. Viral recombinant breakpoints have been suggested to occur in various sites of these regions. In addition, BLAST analysis of the recombinant sequences detected in this study in Japan revealed their high identity with those of viruses detected in the United States and Germany. These deposited samples in Genebank were collected during December 2021 to January 2022, the period of Delta and Omicron variant cocirculation in these countries. Thus, these results suggest that various recombinants are generated in patients with coinfection, particularly during periods of contemporaneous cocirculation of different variants.

Viral recombination, including that of SARS‐CoV‐2, generates new variants with unpredictable epidemic or pathogenic characteristics; in particular, the recombination of Delta and Omicron variants may lead to the acquisition of immune evasion capabilities [26]. Recombinant XD originating from Delta and Omicron variants was identified. However, this strain was transmitted within a local area only and did not spread more extensively [21, 27]. In contrast, XBB sublineages, such as XBB1.16 and EG.5.1, are currently the primary variants circulating worldwide, according to GISAID (https://gisaid.org/). Furthermore, XBB.1.16 has several advantages over other variants, such as a relatively effective reproduction number (R_e_) and resistance to neutralization antibodies [28]. These properties could enable XBB sublineages to circulate worldwide and outcompete previous recombinants. In addition, the number of recombinants identified has rapidly increased, possibly due to high levels of cocirculation between Delta and BA.1 or among Omicron subvariants, and improved genomic surveillance systems in several countries since 2022 [26, 29]. Currently, various Omicron subvariants are circulating worldwide. New recombinants may be generated in patients coinfected with different subvariants. Thus, future emerging SARS‐CoV‐2 strains, including recombinants from coinfected patients that develop into new circulating strains, should be monitored carefully, particularly during periods of cocirculation of multiple variants.

This study has a few limitations. First, we could not conduct variant analysis in six of the 11 patients in the household with SARS‐CoV‐2 infection owing to lack of available samples. Moreover, multiple samples could not be collected from the patient with coinfection; thus, we could not confirm any changes over the course of the infection. The recombination analysis in this study was limited to the full‐length spike gene. Previous studies have reported recombination breakpoints not only in the spike gene, but also in open‐reading‐frame 1a and other regions [23, 26]. Additionally, different viral recombinant sequences were detected by each of the two employed sequencing methods. Future studies should analyze changes in the virus population and recombination in samples from coinfected patients using other genes and sequencing methods (long‐read and Sanger sequencing).

The present study demonstrated the generation of at least nine different viral recombinants in a patient coinfected with the Delta and Omicron variants. The detected recombinants showed a high identity with the virus collected during the cocirculation period in other areas. Currently, various strains, including Omicron subvariants (such as JN.1), are in cocirculation worldwide; thus, novel recombinants in addition to the XBB lineage may be generated. The prevalence of recombinant virus strains, such as XDK and XDD, originating from the JN.1 and XBB/EG.5. sublineages, respectively, has increased worldwide, especially in Europe (GISAID). Thus, the effective genomic surveillance of SARS‐CoV‐2 is also of great clinical importance because viral recombinants can generate viral strains with unpredictable infectivity, virulence, and immune escape characteristics.

## Author Contributions

**Yusuke Sayama:** conceptualization, data curation, formal analysis, funding acquisition, investigation, visualization, writing–original draft preparation, writing–review and editing. **Akie Sakagami:** data curation, formal analysis, investigation, resources, writing–review and editing. **Michiko Okamoto:** project administration, writing–review and editing. **Masahiro Sakamoto:** investigation. **Hikari Koizumi:** investigation, resources. **Yoko Kimura:** investigation, resources. **Clyde Dapat:** writing–review and editing. **Mayuko Saito:** writing–review and editing. **Yuko Suzuki:** investigation, resources. **Mie Sasaki:** investigation, resources. **Naoko Sugawara:** investigation, resources. **Hitoshi Oshitani:** funding acquisition, writing–review and editing; supervision.

## Ethics Statement

This study was approved by the Tohoku University Ethics Review Board (#2022‐1‐580).

## Conflicts of Interest

The authors have no conflict of interest to declare.

## Supporting information


**Figure S1.** Description of viral quasispecies nucleotides obtained in this study using long‐read and Sanger sequencing.
**Figure S2.** Description of viral quasispecies amino acids obtained in this study using long‐read and Sanger sequencing.
**Figure S3.** SimPlot analysis for putative SARS‐CoV‐2 recombinants obtained based on Sanger sequencing.
**Figure S4.** SimPlot analysis of detected SARS‐CoV‐2 strains to determine similarity with other viruses using BLAST analysis.
**Table S1.** Results of a BLAST search using recombinant sequences obtained from this study.

## Data Availability

The raw sequence data generated from each sample in this study have been deposited in the DNA Data Bank of Japan/European Molecular Biology Laboratory/GenBank (DRR438334 to DRR438339).
