# Supplementary material for: Identification of Various Recombinants in a Patient Coinfected With the Different SARS‐CoV‐2 Variants
Source: Influenza Other Respir Viruses. 2024 Jun 18;18(6):e13340. doi: 10.1111/irv.13340 (PMC11187932; doi:10.1111/irv.13340)
Supplement: Supplementary file 1 — Figure S1. Description of viral quasispecies nucleotides obtained in this study using long‐read and Sanger sequencing. Figure S2. Description of viral quasispecies amino acids obtained in this study using long‐read and Sanger sequencing. Figure S3. SimPlot analysis for putative SARS‐CoV‐2 recombinants obtained based on Sanger sequencing. Figure S4. SimPlot analysis of detected SARS‐CoV‐2 strains to determine similarity with other viruses using BLAST analysis. Table S1. Results of a BLAST search using recombinant sequences obtained from this study. [file IRV-18-e13340-s001.docx]

**Supplementary materials**

**Identification of various recombinants in a patient co-infected with the different SARS-CoV-2 variants**


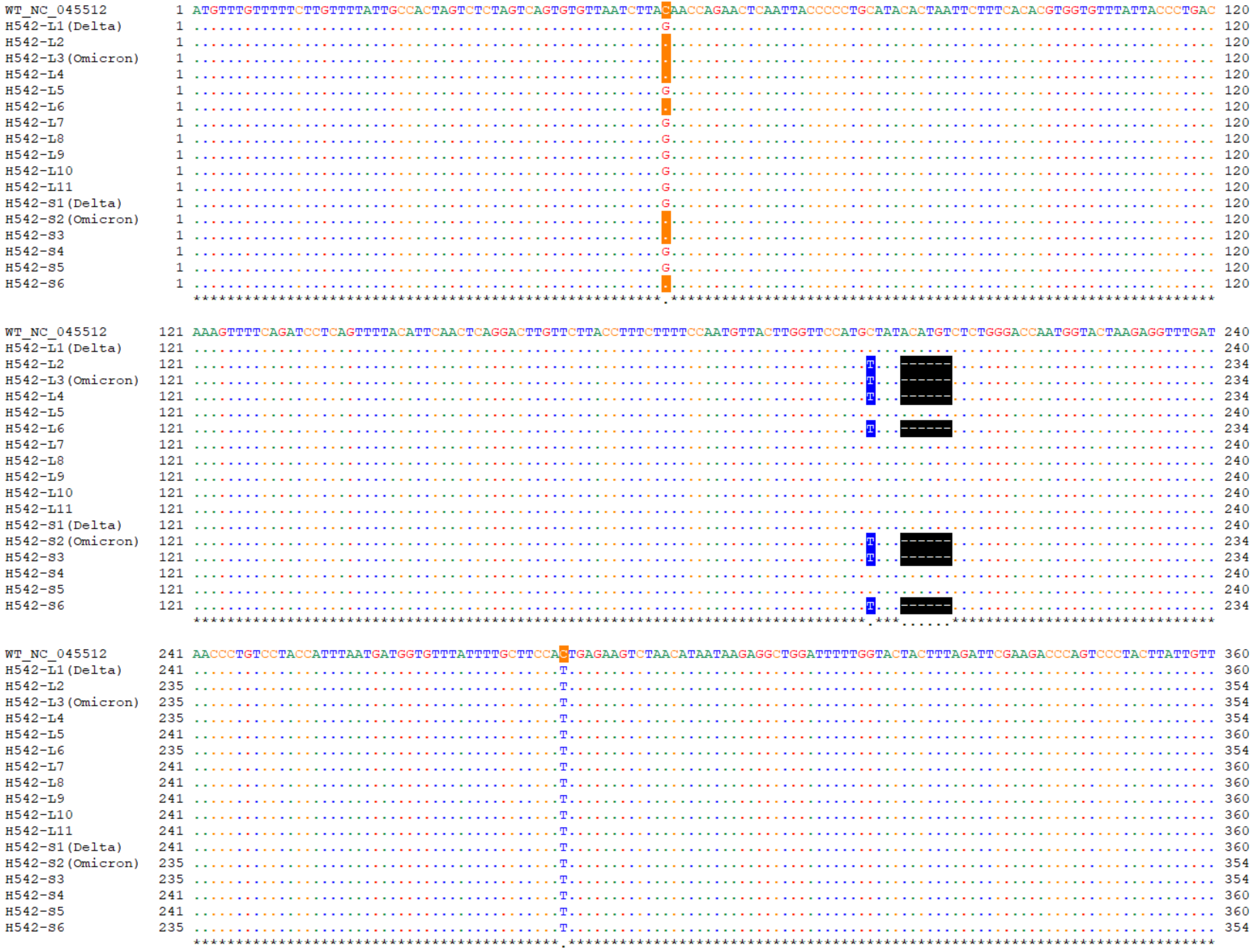


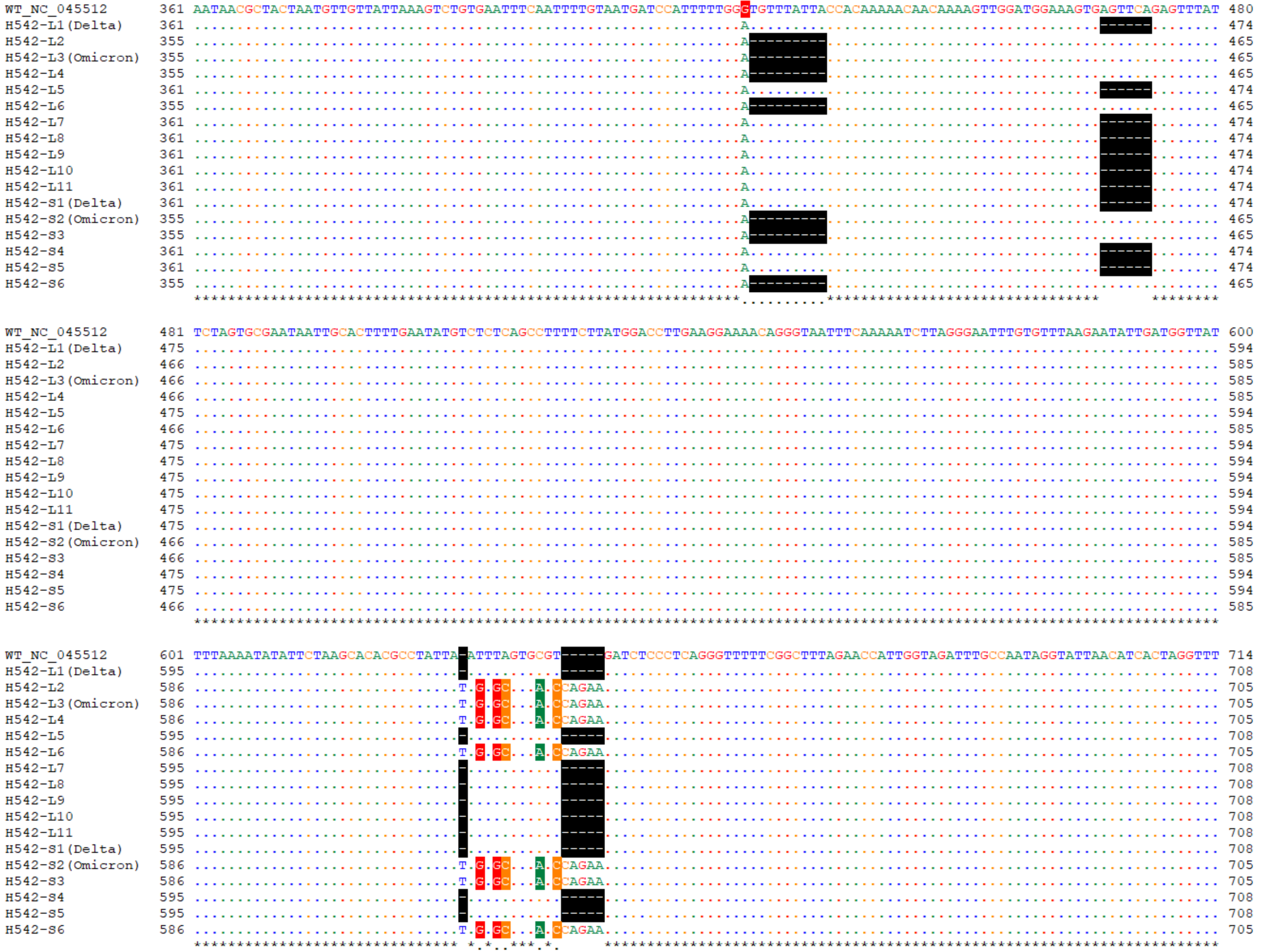


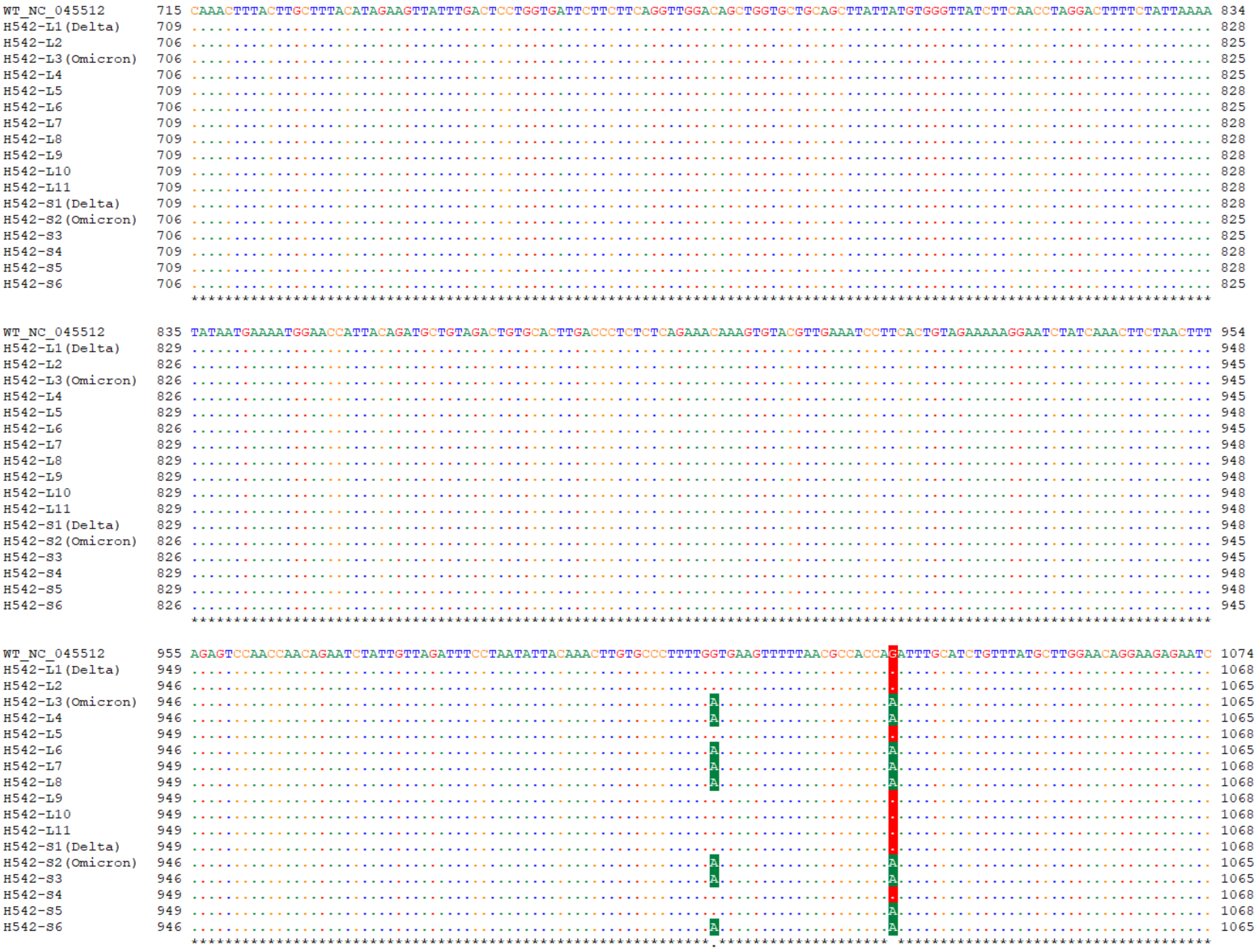


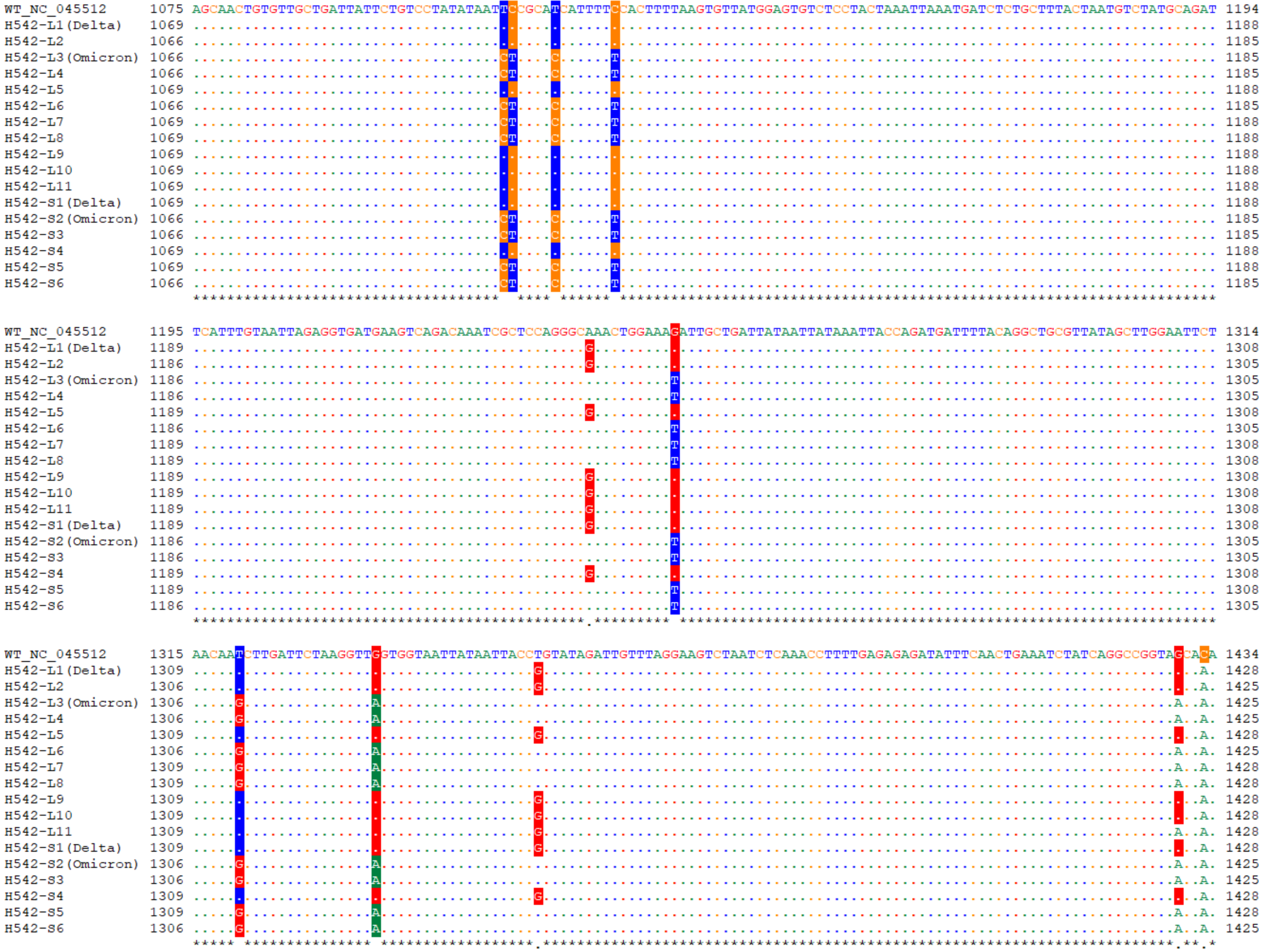


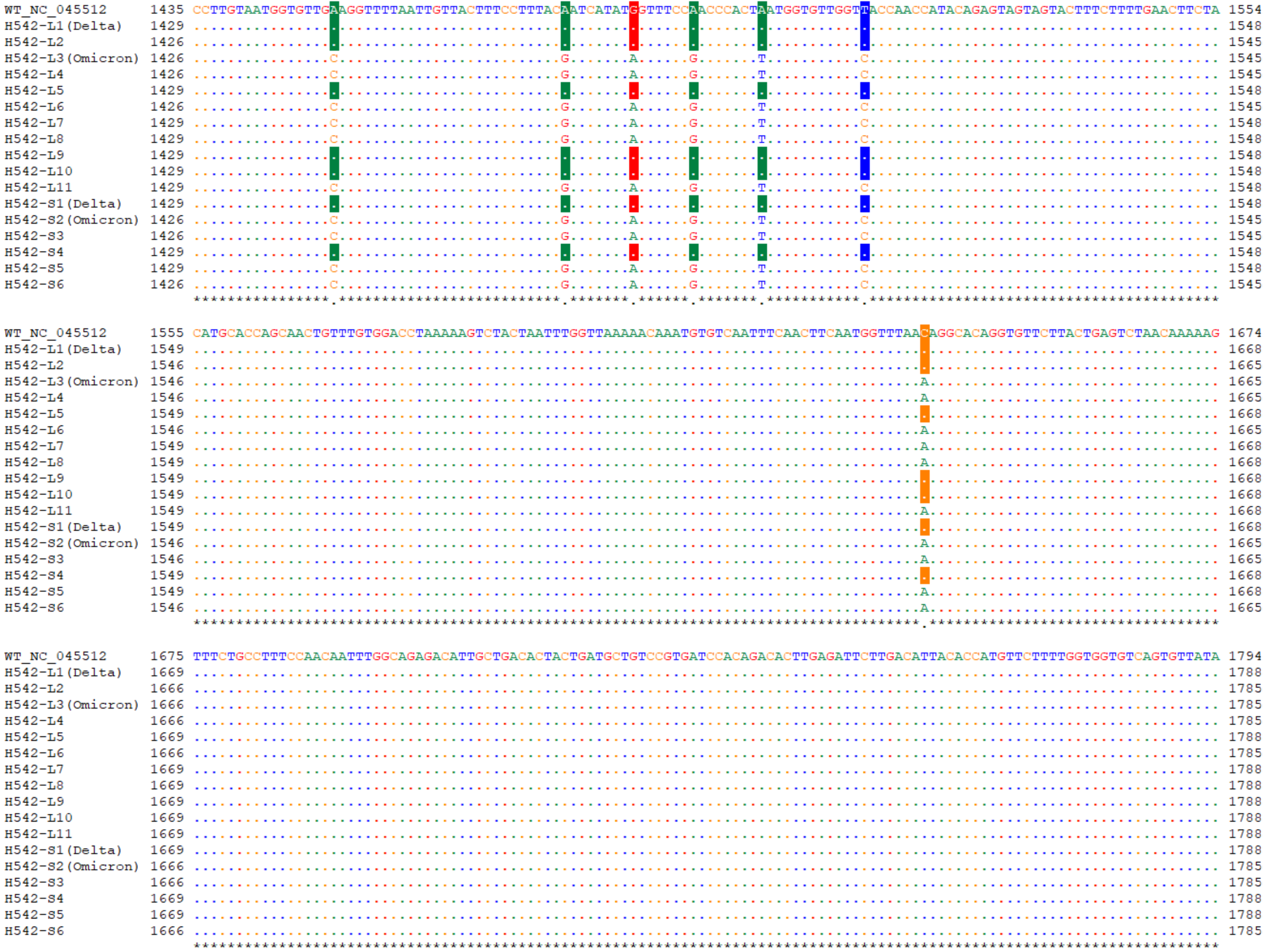


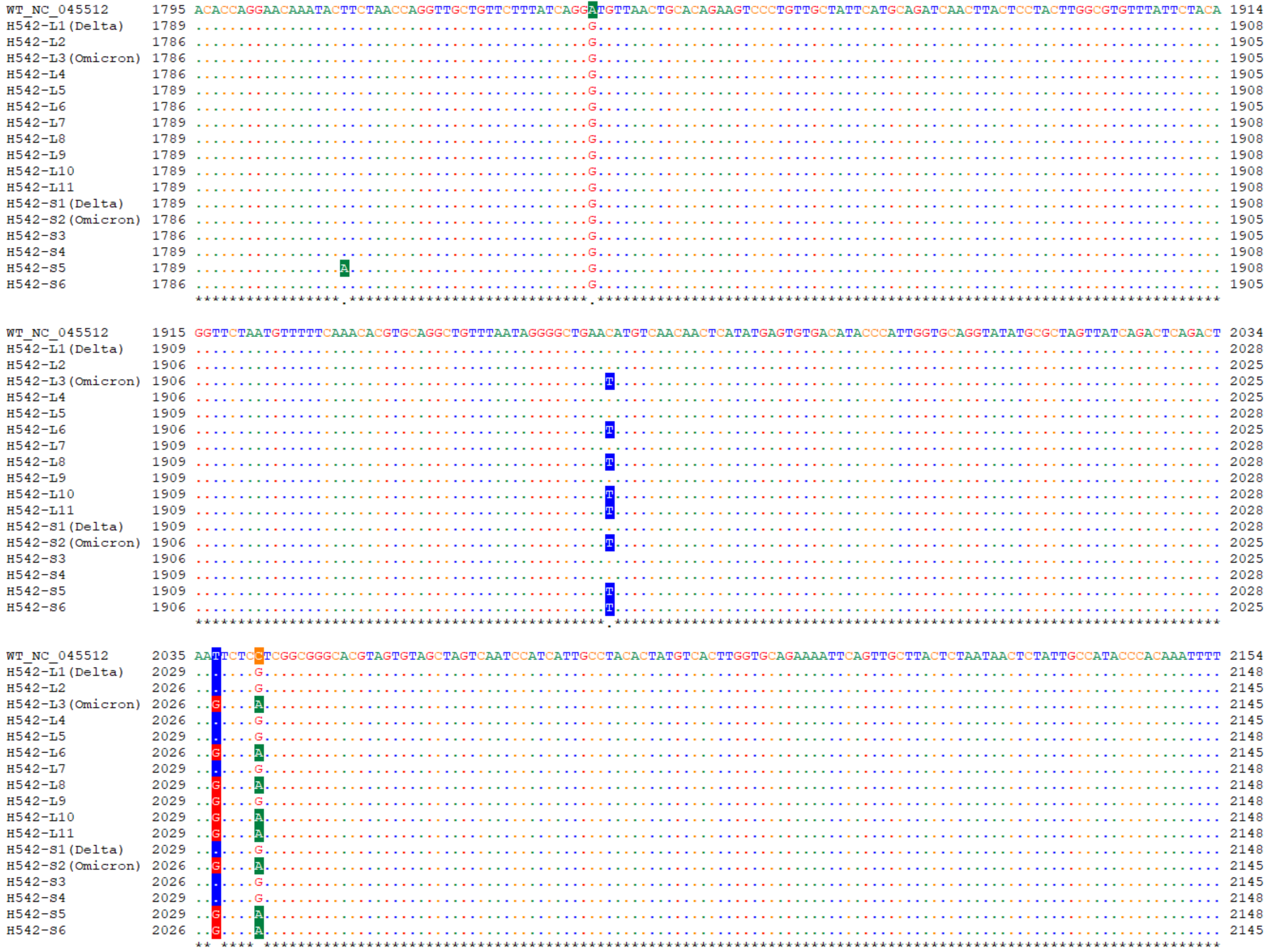


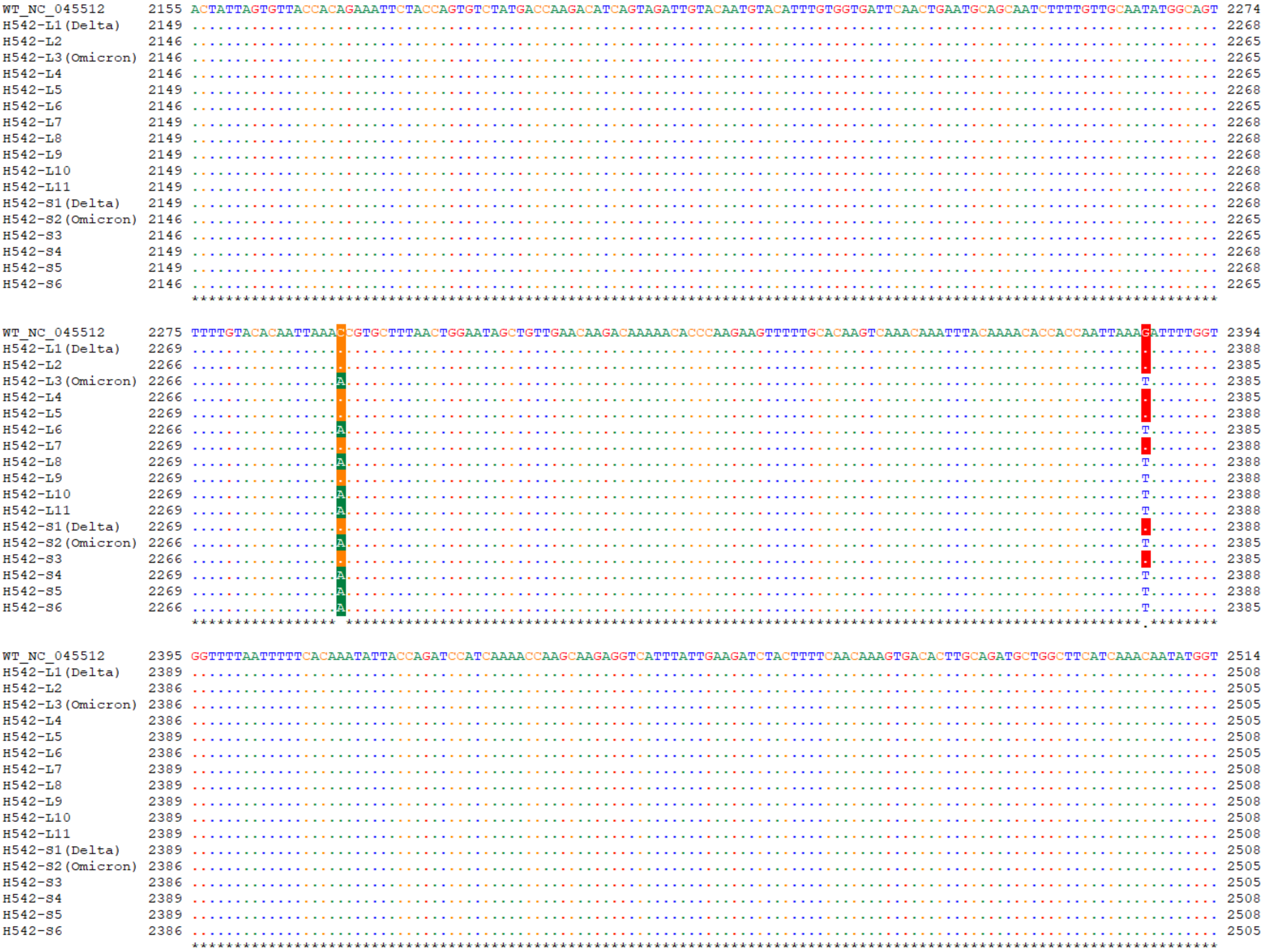


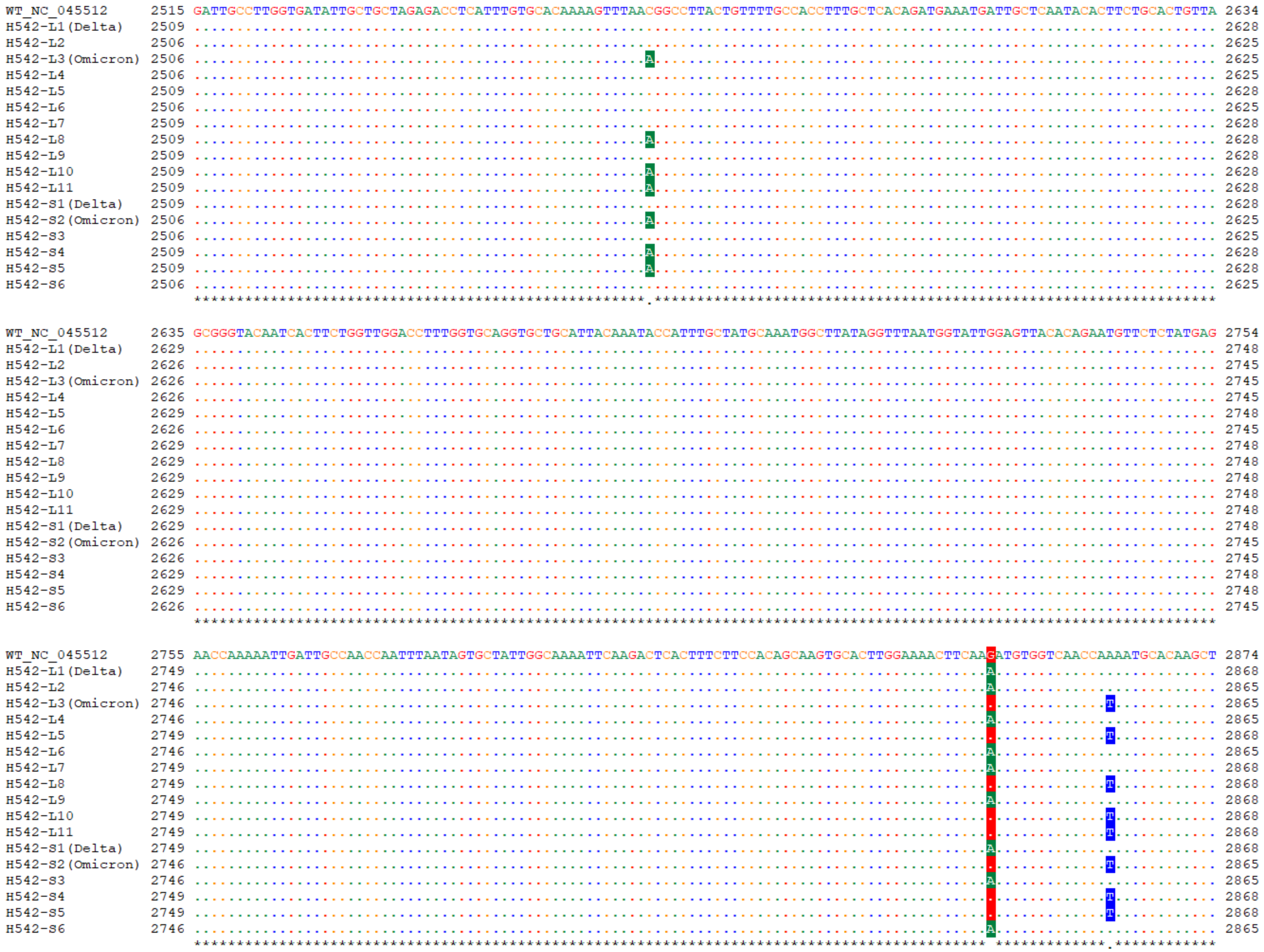


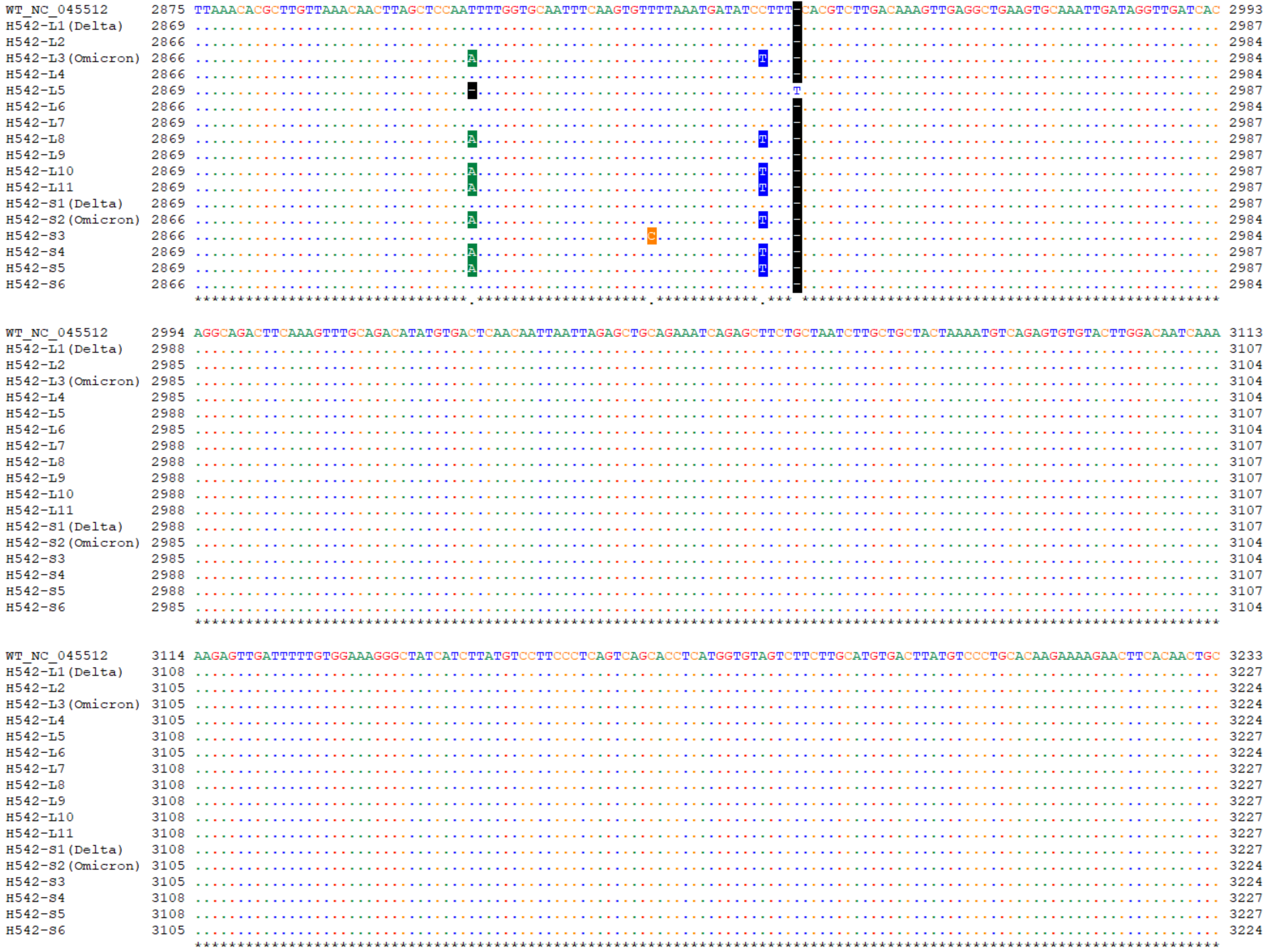


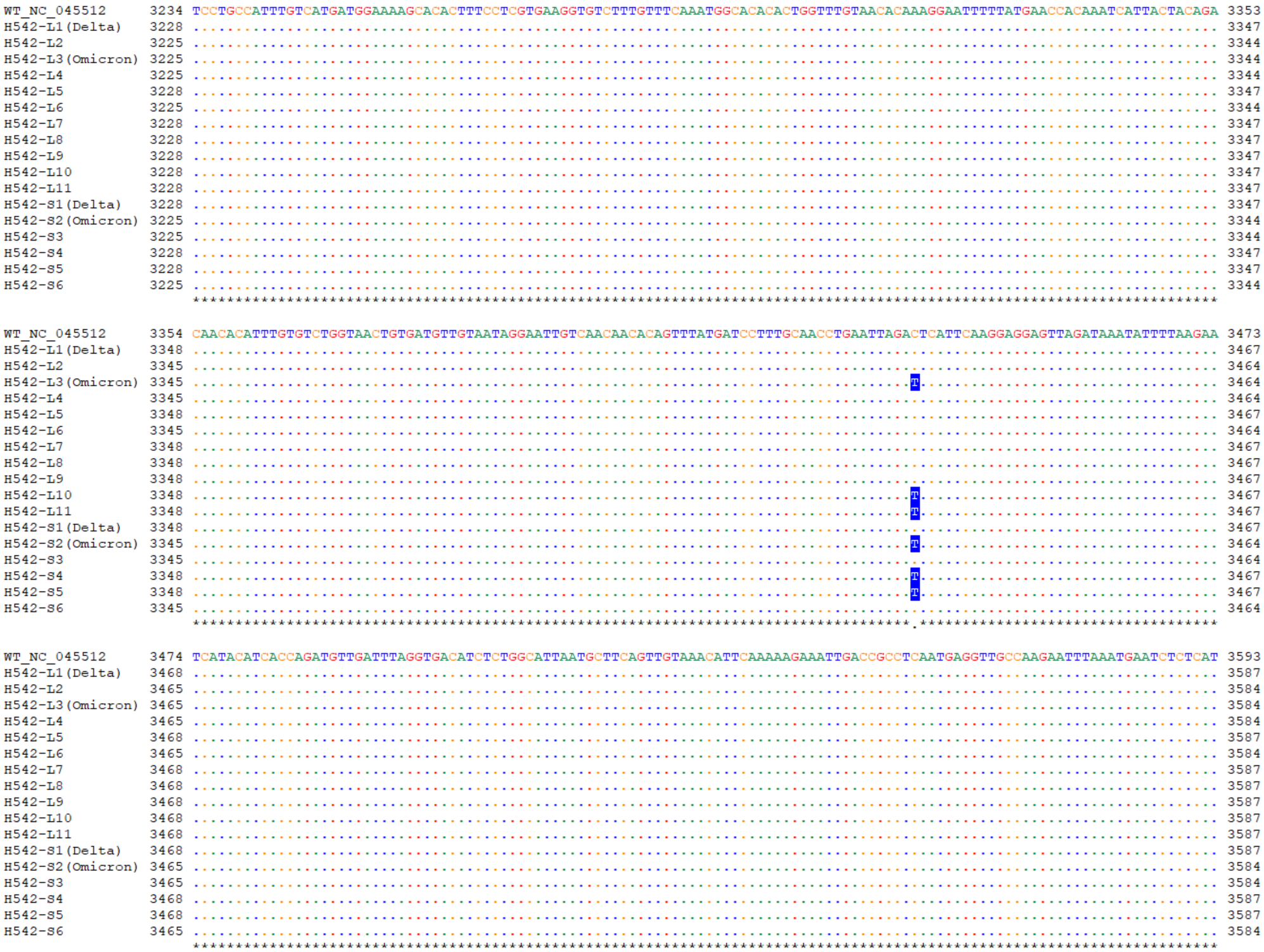


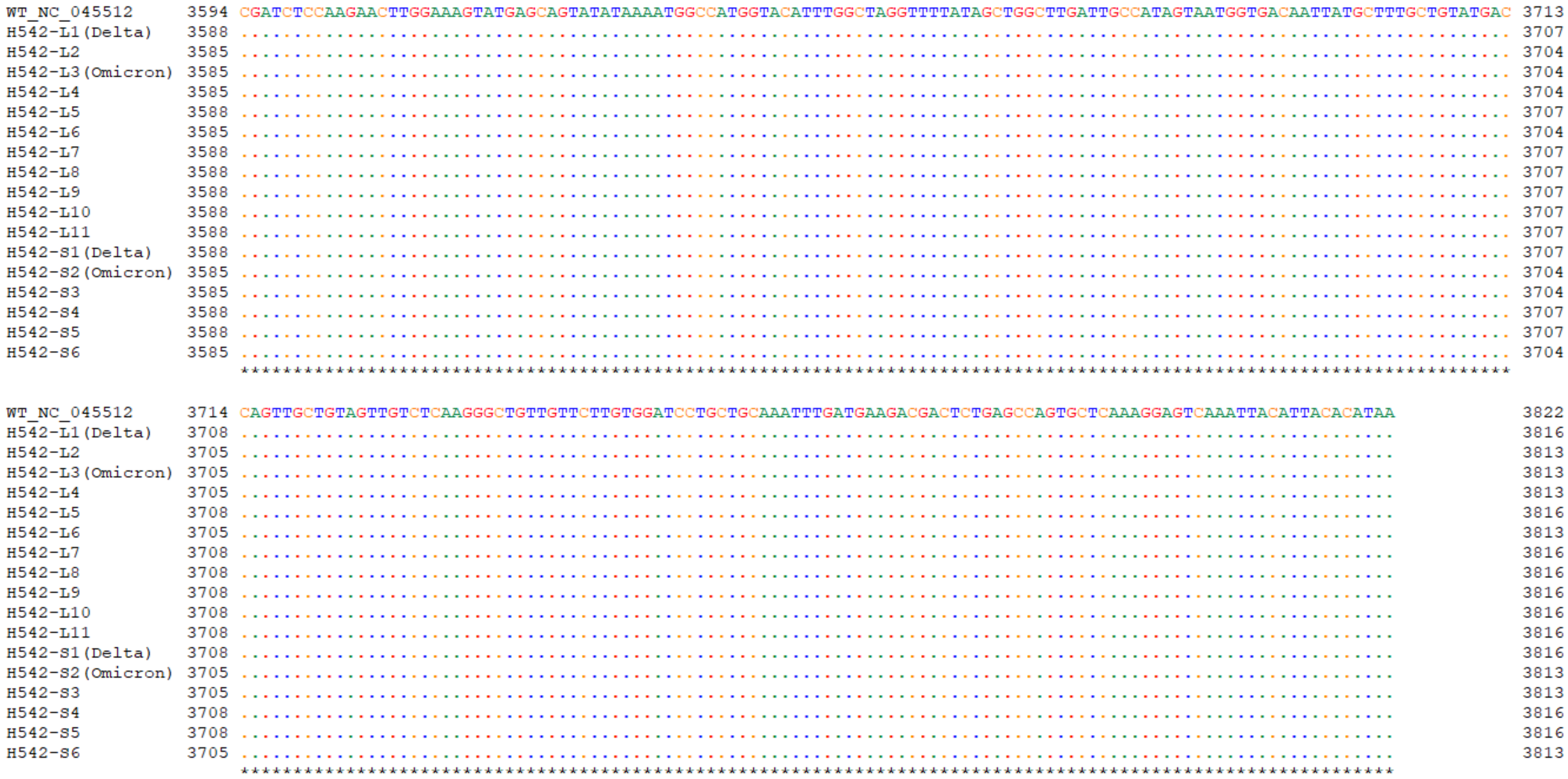


**Figure S1. Description of viral quasispecies nucleotides obtained in this study using long-read and Sanger sequencing.** H542-L1 to L11 obtained based on long-read sequencing. H542-S1 to S6 obtained based on Sanger sequencing. A mismatch is highlighted. Reference strain: WT strain (Accession no. NC_045512).


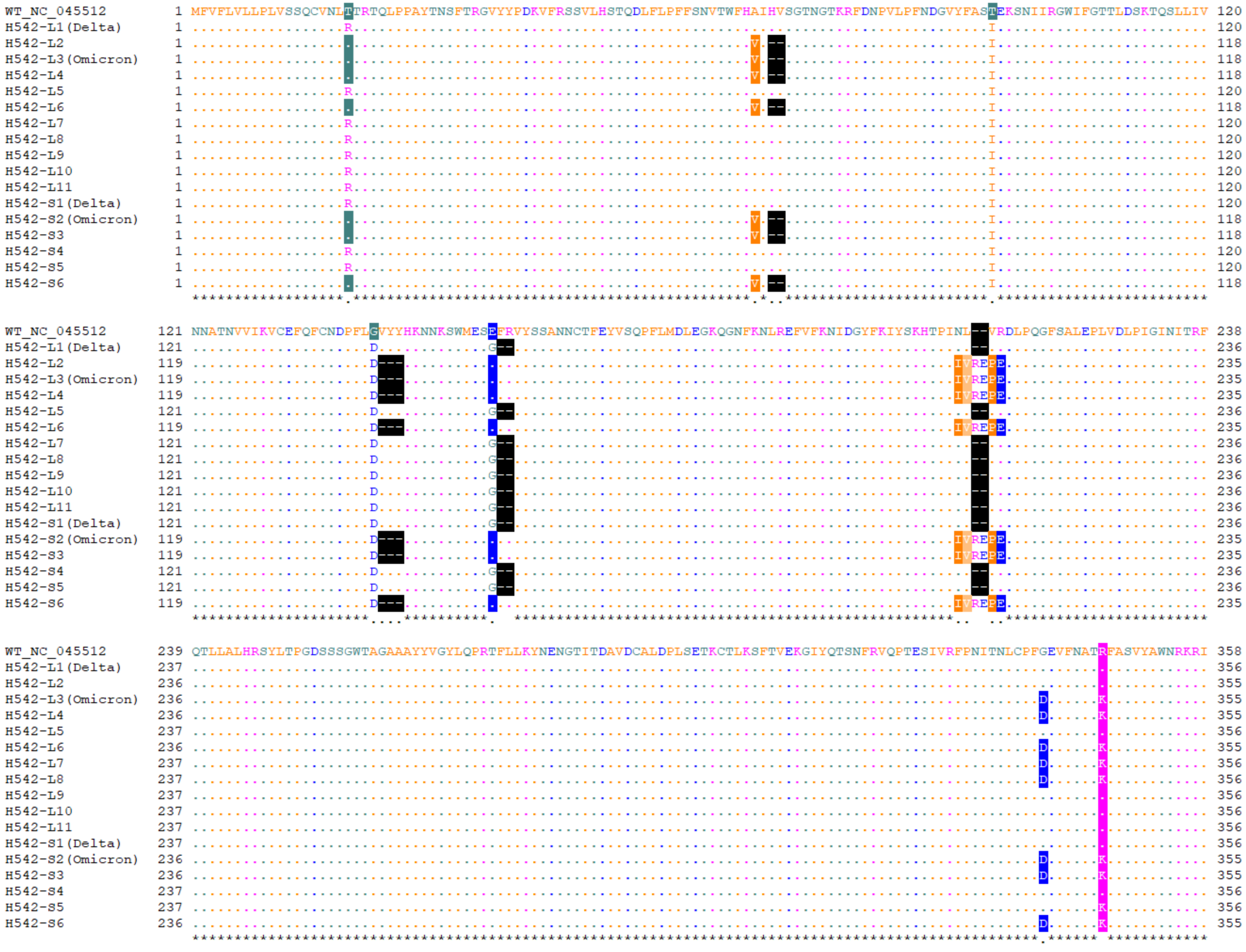


**
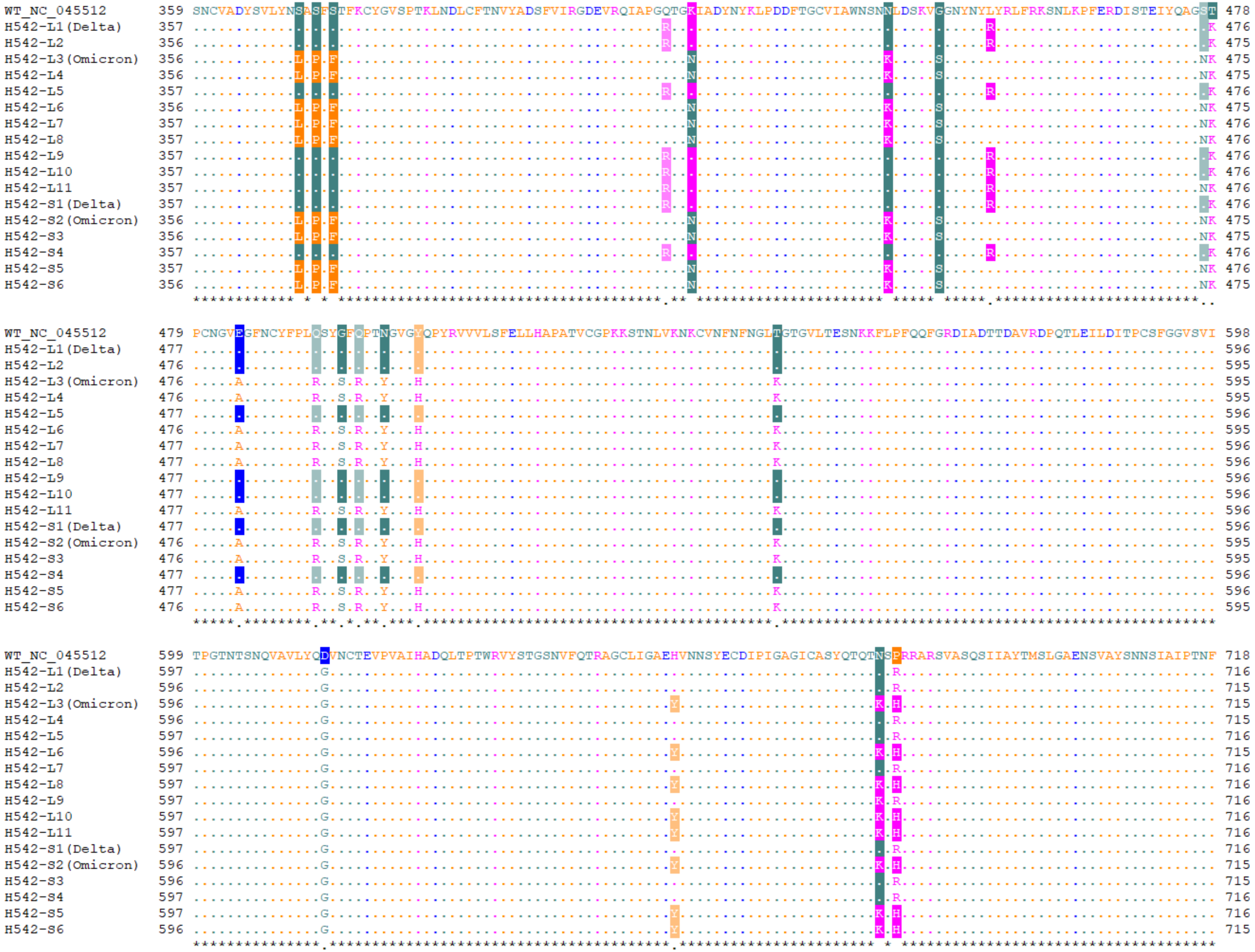
**

**
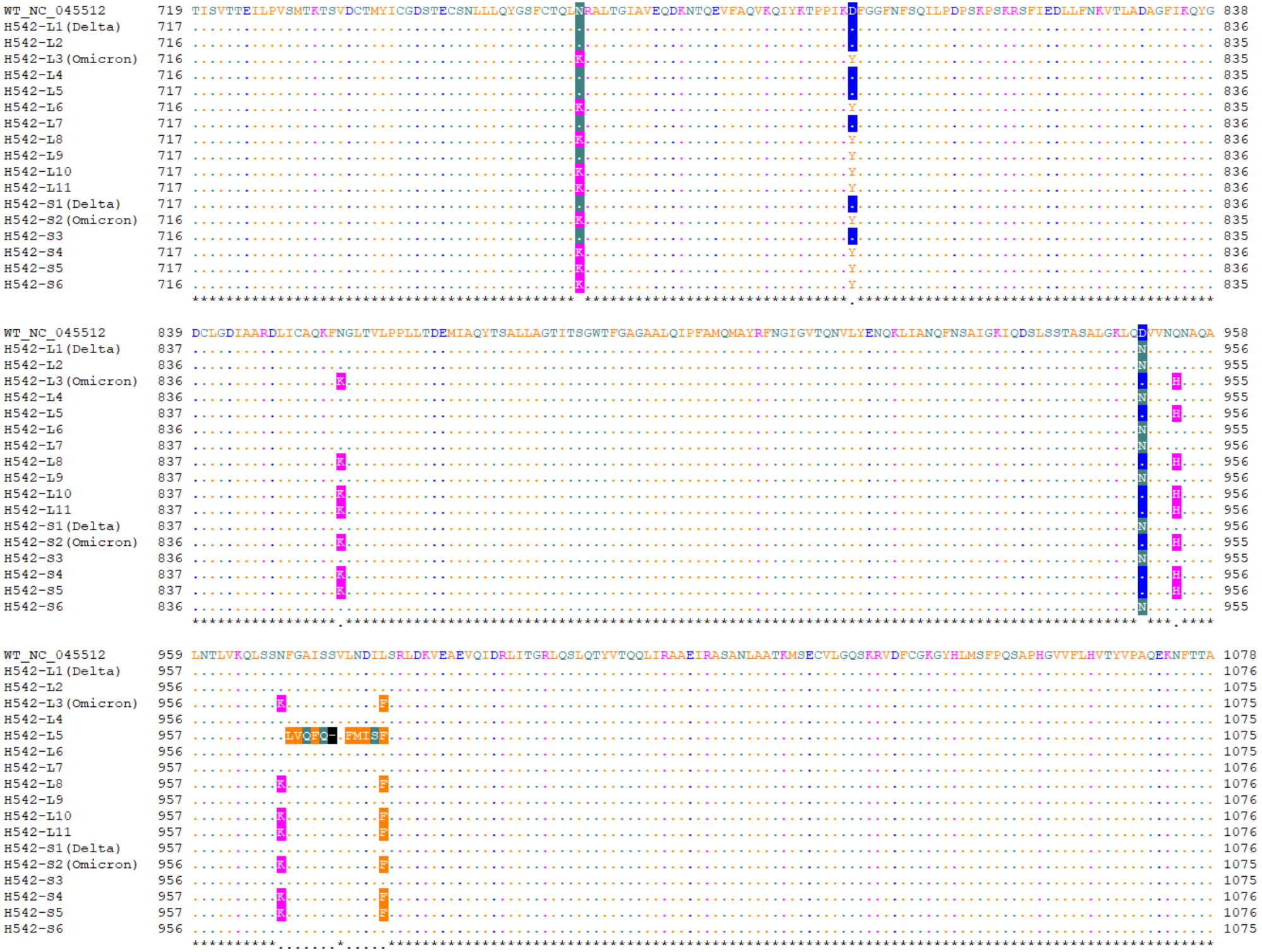
**

**
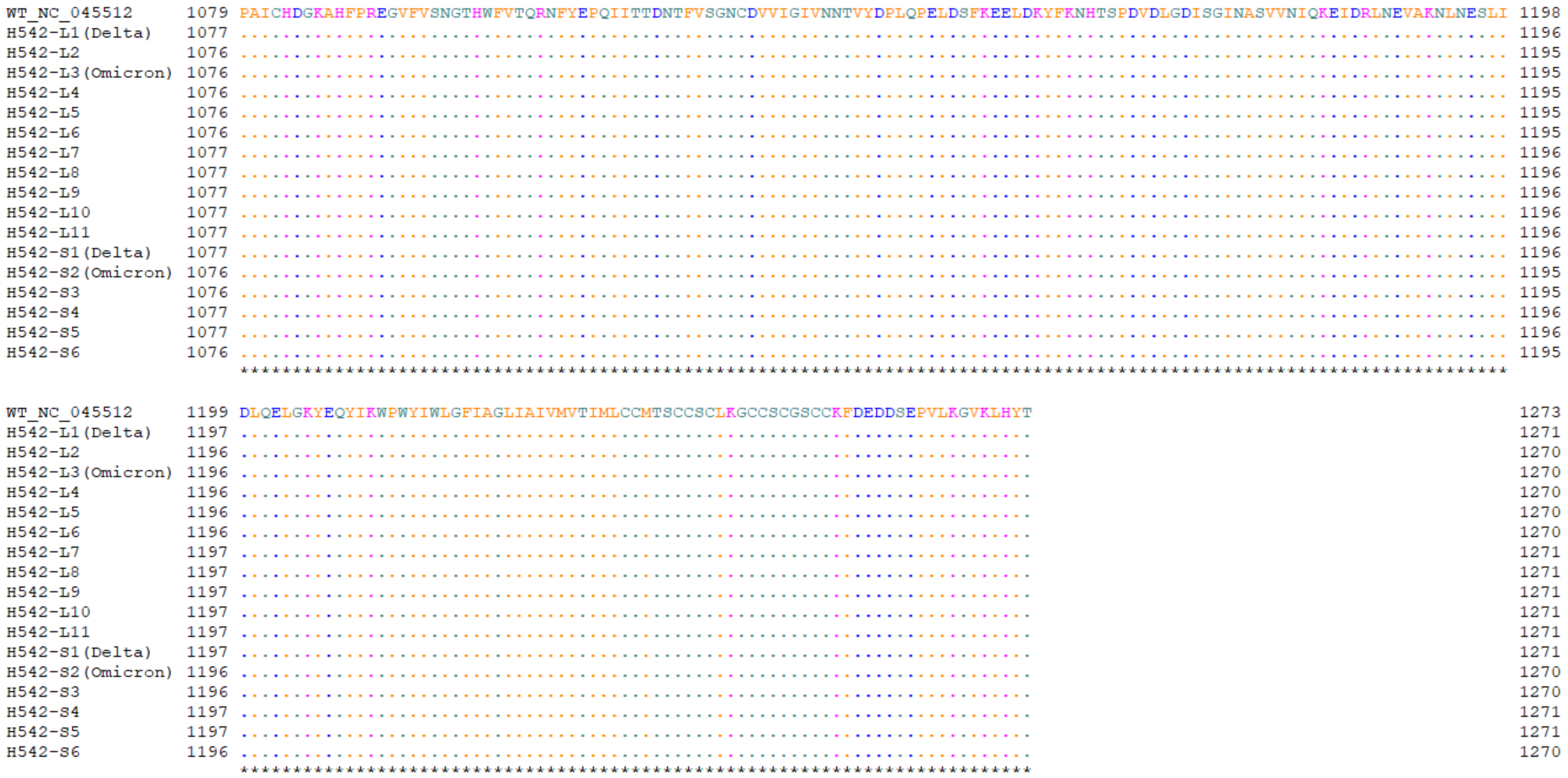
**

**Figure S2. Description of viral quasispecies amino acids obtained in this study using long-read and Sanger sequencing.** H542-L1 to L11 obtained based on long-read sequencing. H542-S1 to S6 obtained based on Sanger sequencing. A mismatch is highlighted. Reference strain: WT strain (Accession no. NC_045512).


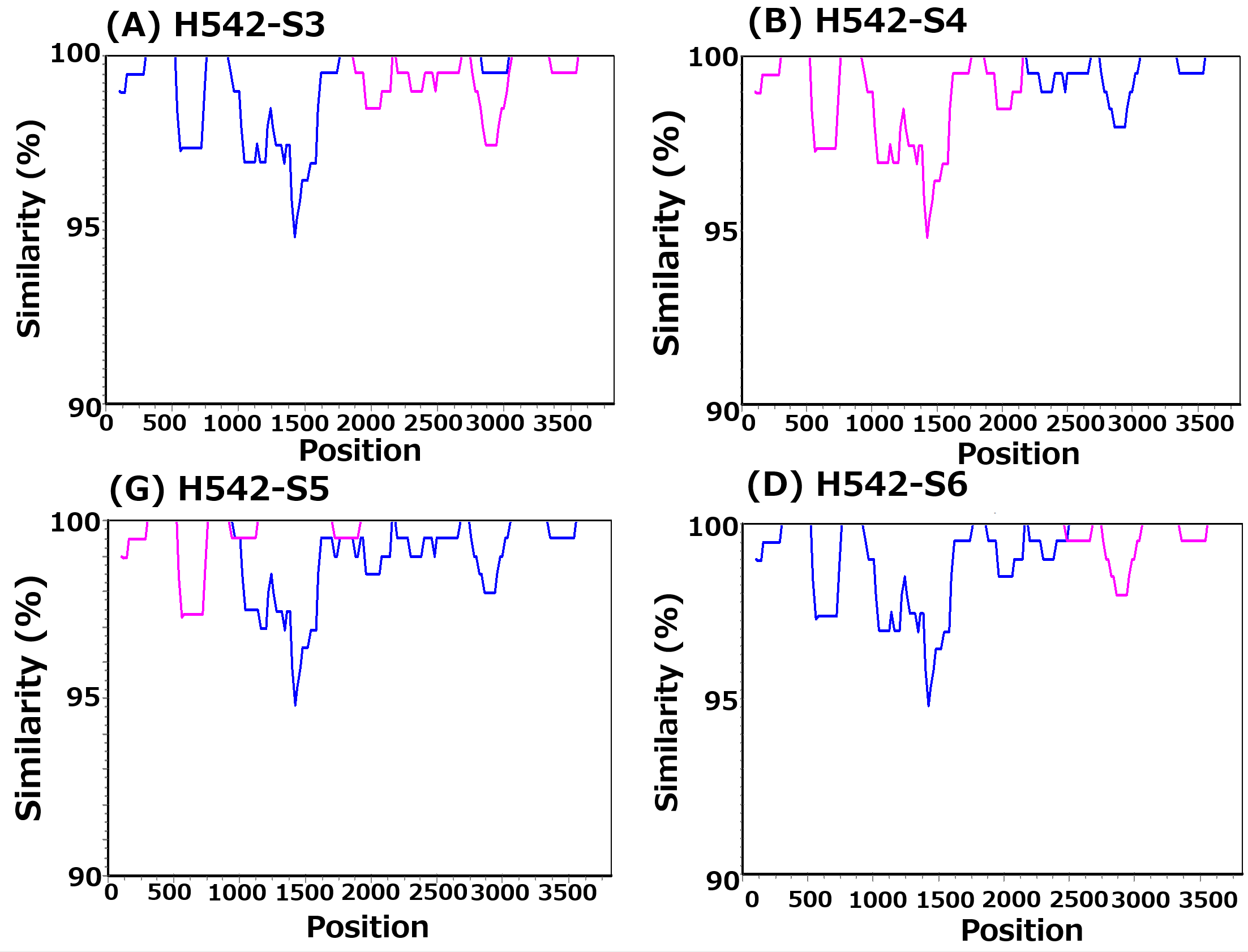


**Figure S3. SimPlot analysis for putative SARS-CoV-2 recombinants obtained based on Sanger sequencing.** Comparisons of genetic similarity between recombinant and Delta (H542-S1/blue) and Omicron (H542-S2/magenta) sequences were made using the SimPlot software. The results are shown for the viral quasispecies sequences H542-S3 (A), H542-S4 (B), H542-S5 (C), and H542-S6 (D). The vertical axis represents the percent nucleotide sequence similarity between the putative recombinant and each strain used for comparison, and the horizontal axis shows the relative nucleotide position along the Spike gene. For each analysis, a window size of 200 nucleotides and the Kimura distance model (2-parameter) were used.


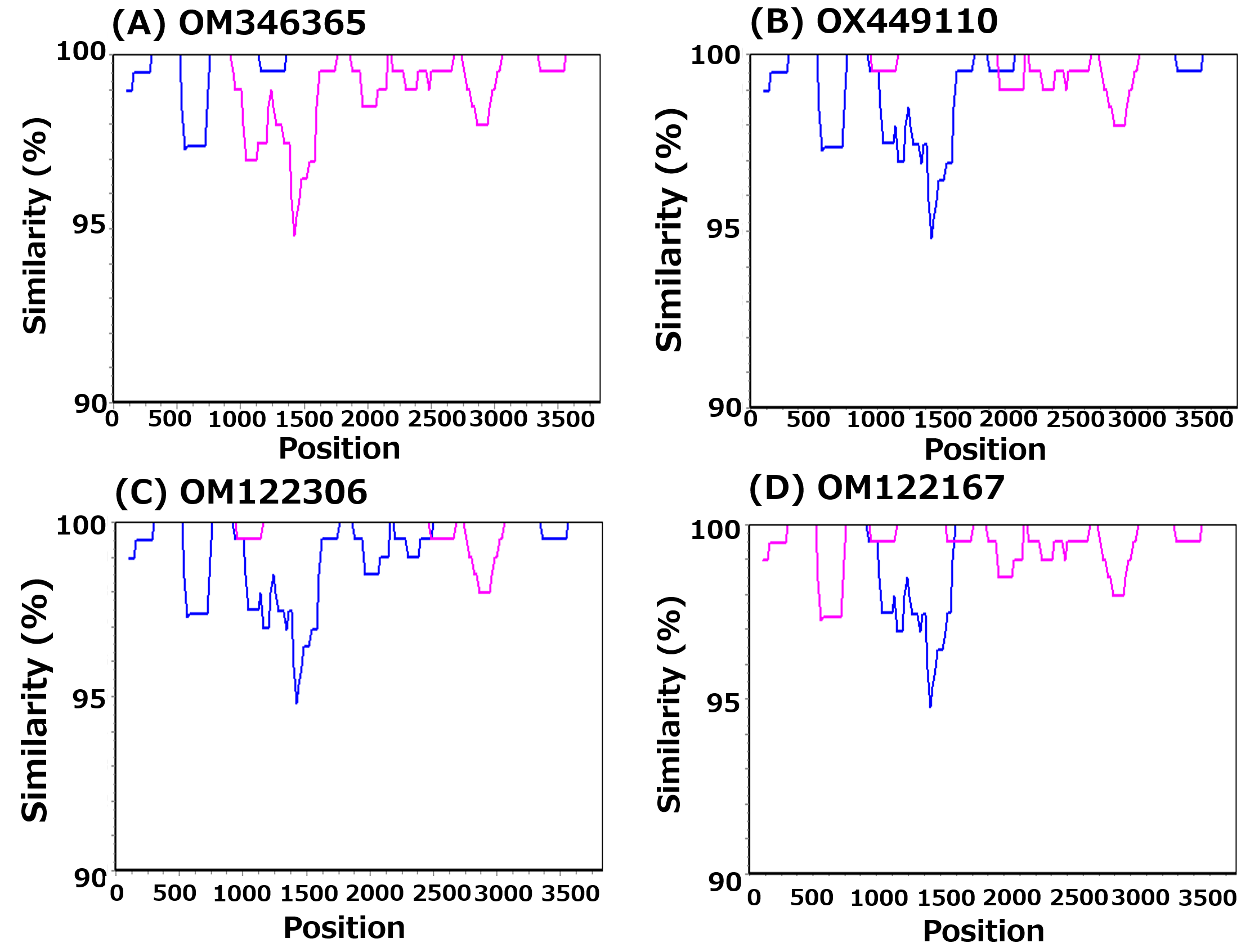


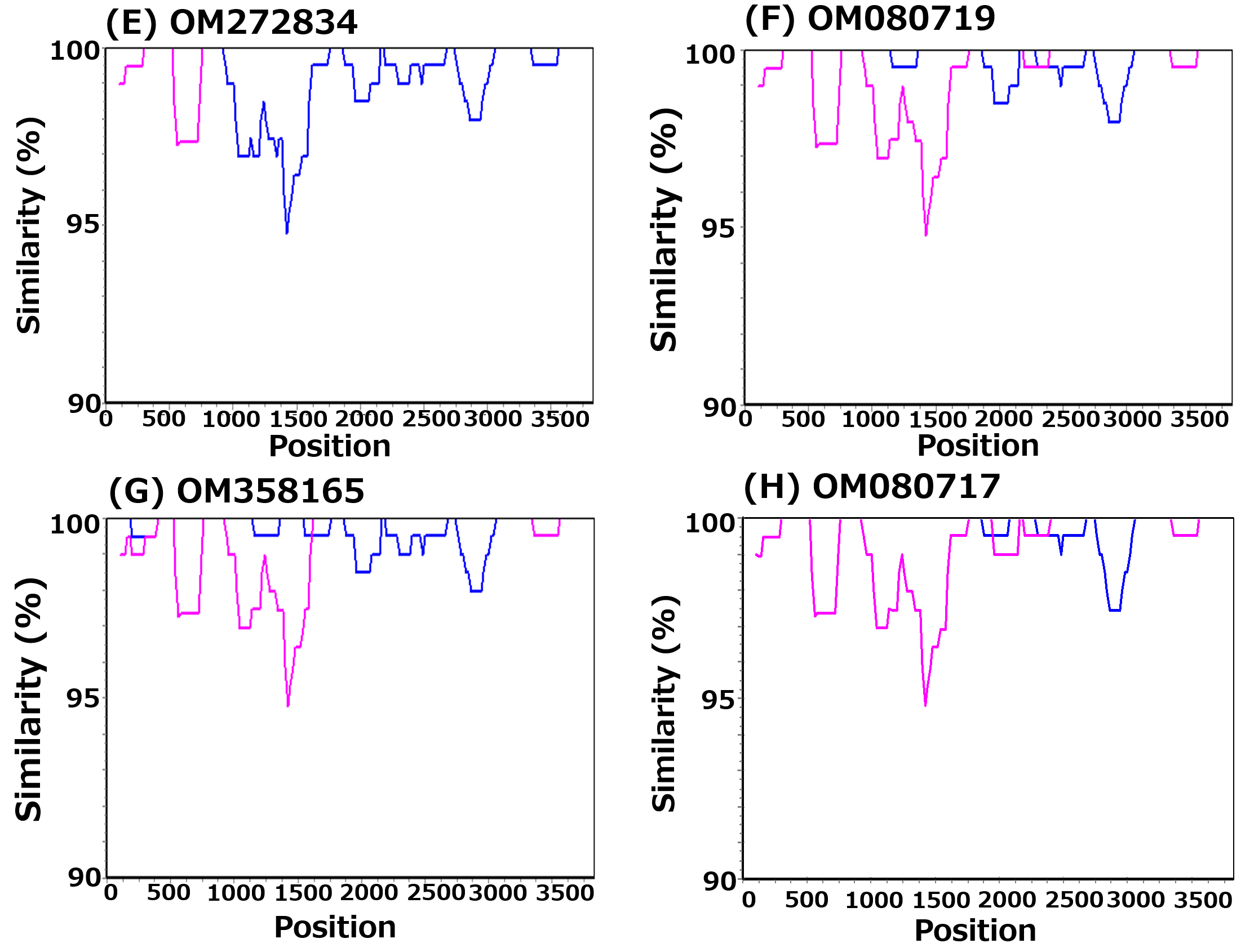


**Figure S4. SimPlot analysis of detected SARS-CoV-2 strains to determine similarity with other viruses using BLAST analysis.**

Comparisons of genetic similarity between detected viral sequence and Delta (H542-L1/blue) and Omicron (H542-L3/magenta) strains were made using the SimPlot software. The results are shown for the detected viral sequences OM346365 (A), OX449110 (B), OM122167 (C), OM122167 (D), OM272834 (E), OM080719 (F), OM358165 (G), and OM080717 (H). The vertical axis represents the percent nucleotide sequence similarity between the putative recombinant and each strain used for comparison, and the horizontal axis shows the relative nucleotide position along the Spike gene. For each analysis, a window size of 200 nucleotides and the Kimura distance model (2-parameter) were used.

**Table S1. Results of a BLAST search using recombinant sequences obtained from this study**

| ID | Strain name | Accession No. | Geographic location | Homology (%) | Pangolin | Collection date |
| --- | --- | --- | --- | --- | --- | --- |
| L542-L2 | SARS-CoV-2/human/USA/MA-CDCBI-CRSP_XJWNE7BGY7HQE4BA/2022 | OM346365 | USA (Massachusetts) | 3812/3813 (99.97) | B.1.617.2 | Jan 10, 2022 |
| L542-L4 | 20220201855 | OX449110 | Germany | 3810/3813 (99.92) | BA.1.15 | Jan 13, 2022 |
| L542-L6 | SARS-CoV-2/human/USA/DC-CDC-LC0438060/2021 | OM122306 | USA (District of Columbia) | 3811/3813 (99.95) | BA.1 | Dec 21, 2021 |
| L542-L7 | SARS-CoV-2/human/USA/NC-CDC-LC0438130/2021 | OM122167 | USA (North Carolina) | 3814/3816 (99.95) | BA.1 | Dec 21, 2021 |
| L542-L8 | SARS-CoV-2/human/USA/TN-CDC-ASC210559252/2021 | OM272834 | USA (Tennessee) | 3815/3816 (99.97) | BA.1.1 | Dec 31, 2021 |
| L542-L10 | SARS-CoV-2/human/USA/MN-CDC-IBX761934107699/2021 | OM080719 | USA (Minnesota) | 3813/3816 (99.92) | B.1.617.2 | Dec 8, 2021 |
| L542-L11 | SARS-CoV-2/human/USA/ID-CDC-LC0467673/2022 | OM358165 | USA (Idaho) | 3806/3816 (99.74) | B.1.617.2 | Jan 2, 2022 |
| L542-S3 | 20220201855 | OX449110 | Germany | 3809/3813 (99.90) | BA.1.15 | Jan 13, 2022 |
| L542-S4 | SARS-CoV-2/human/USA/MN-CDC-IBX745157306861/2021 | OM080717 | USA (Minnesota) | 3812/3816 (99.90) | AY.44 | Dec 8, 2021 |
| L542-S5 | SARS-CoV-2/human/USA/TN-CDC-ASC210559252/2021 | OM272834 | USA (Tennessee) | 3814/3816 (99.95) | BA.1.1 | Dec 31, 2021 |
| L542-S6 | SARS-CoV-2/human/USA/DC-CDC-LC0438060/2021 | OM122306 | USA (District of Columbia) | 3811/3813 (99.95) | BA.1 | Dec 21, 2021 |

*Geographic location, Homology, Pangolin, and Collection date were obtained from the NCBI data bank.
